# Pupal Development and Adult Acclimation Temperatures Influence the Cold and Heat Tolerance in *Tenebrio molitor* (Coleoptera: Tenebrionidae)

**DOI:** 10.3390/insects16040402

**Published:** 2025-04-11

**Authors:** Jan Podlesnik

**Affiliations:** Faculty of Natural Sciences and Mathematics, University of Maribor, 2000 Maribor, Slovenia; jan.podlesnik@um.si

**Keywords:** adult acclimation, cold tolerance, developmental acclimation, heat knockdown, heat tolerance, *Tenebrio molitor*, thermal tolerance, chill coma, yellow mealworm

## Abstract

Temperature plays a crucial role in shaping the biology of insects. Developmental temperature and acclimation temperatures influence their ability to cope with extreme thermal conditions. This study investigates the effects of developmental temperatures during the pupal stage and adult acclimation temperatures on the thermal tolerance of *Tenebrio molitor* Linnaeus, 1758. We investigated cold tolerance based on chill-coma recovery time and heat tolerance based on heat knockdown time. Beetles were reared at five developmental temperatures (16, 21, 25, 30 and 35 °C) and later exposed to corresponding adult acclimation temperatures. From developmental temperatures of 21 and 30 °C, the group of beetles was subjected to different temperatures to induce adult acclimation at a different temperature than the developmental temperature. In cold-tolerance tests, beetles reared at lower temperatures showed better recovery from chill coma, while beetles reared at higher temperatures showed greater resistance to heat shock. Adult beetles acclimated to lower temperatures showed better cold tolerance, while those acclimated to higher temperatures performed better in the heat tolerance test. Interestingly, the developmental temperatures during the pupal stage also contributed to resistance, particularly in the heat-tolerance test. However, pupal stage temperatures had no effect on cold-shock resistance, as indicated by chill-coma recovery time. The results could provide insights into the rearing of *T. molitor.*

## Simple Summary

This study examines how developmental and adult temperature exposures affect the cold and heat tolerance of *Tenebrio molitor* Linnaeus, 1758, revealing that adult acclimation plays a more significant role in thermal resilience. In addition, developmental temperatures also show a significant influence on heat tolerance. The results improve our understanding of the mechanisms of insect acclimation and provide insights into the management of *T. molitor* in different ecological and economic contexts.

## 1. Introduction

Temperature is a key abiotic factor that influences every aspect of insect biology. It affects metabolic rates and thermal developmental limits, developmental rates and response, as well as an insect’s response to unfavourable conditions. As a result, insects often show rapid physiological adjustments to thermal changes, which are typically reversible [1,2]. The genotype of an individual plays a critical role in providing solutions to different environmental conditions, while varying environmental conditions shape the phenotype through gene expression [3]. This phenomenon is known as phenotypic plasticity, which enables organisms to cope effectively with environmental changes or new environments.

One manifestation of phenotypic plasticity is acclimation, which refers to an organism’s physiological, biochemical or anatomical response to prolonged exposure to a new environmental condition [4]. The term refers to a response to changes in a single abiotic parameter, such as temperature. It can occur over a short period (hours or days) or be developmentally dependent, in which case it is referred to as developmental plasticity or developmental acclimation [5,6,7,8]. In this broader sense, acclimation encompasses processes like hardening, adult acclimation and developmental plasticity.

Hardening refers to short-term exposure to extreme, non-lethal temperatures, which enhances an organism’s resilience to future extreme conditions [9]. The effects of hardening are short-lived, typically lasting minutes to hours. Adult acclimation involves prolonged exposure to a specific temperature, not necessarily extreme, and can last for days to weeks [10,11]. A substantial body of research has demonstrated the positive effects of hardening and adult acclimation on an organism’s ability to withstand extreme temperatures. For instance, as temperatures begin to drop in the fall, insects acclimate to lower sublethal temperatures, preparing them for the winter cold [12,13]. Similarly, hardening, through short-term exposure to extreme temperatures, enables insects to better withstand similar conditions in subsequent exposures [9,14,15].

The third type of acclimation, developmental plasticity (or developmental acclimation), remains more controversial. Research has shown that developmental temperature can influence the fitness of organisms in various ways. The beneficial acclimation hypothesis (BAH) posits that individual’s development at a particular temperature leads to enhanced performance at that same temperature [16]. While BAH is intuitive and some studies provide support for it, it cannot be universally applied [5,17,18,19,20]. Alternative hypotheses on developmental plasticity suggest that intermediate, high or low developmental temperatures may equip organisms with the best performance across the entire temperature range of an organism [5]. Numerous studies have provided evidence for these alternative hypotheses [5,8,17,21,22,23,24,25,26,27,28,29,30].

*Tenebrio molitor* Linnaeus, 1758 (yellow mealworm) is a globally widespread storage pest known for infesting cereal, flour, bran and pasta [31]. In recent years, research interest in *T. molitor* has increased due to its potential as an alternative protein source for animal feed and human nutrition [32,33]. Its use in waste management has also been recognised, making it an ideal candidate for the circular economy [34]. Consequently, its biology is being studied in greater detail [35]. Despite the growing body of research, gaps remain in our understanding of *T. molitor* biology, particularly in the field of thermal biology.

Most research on *T. molitor* thermal biology has focused on determining optimal temperature conditions for rearing. The optimal development temperature for *T. molitor* is reported to be between 25–30 °C [35,36,37], with some studies suggesting 31 °C as the optimal temperature leading to maximum daily growth rates [38] (Bjørge et al. 2018). Thermal limits have also been investigated [39] (Stevens et al. 2010), with temperatures below 17 °C inhibiting embryonic development and temperatures below 10 °C or above 35 °C increasing the mortality rates [36,40,41]. Additionally, previous research has identified minimum and maximum lethal temperatures: the minimum lethal temperature for a 24-hour exposure is 7–8 °C [42], while the maximum lethal temperature ranges from 40 to 44 °C [43]. However, the effects of acclimation on thermal tolerance in *T. molitor* remain largely unexplored. Some studies have examined the rate of temperature change and its impact on acclimation and subsequent thermal limits [44], though these focused solely on adult acclimation. To our knowledge, fundamental effects of acclimation—both developmental and adult—on thermal tolerance in *T. molitor* have not been thoroughly investigated.

Thermal tolerance can be investigated by using chill-coma recovery time and heat knockdown time as measures for cold and heat tolerance. Both are commonly used measures of cold and heat tolerance in insects [45,46]. When exposed to low temperatures, many insect species enter a reversible comatose state, known as a chill coma [47]. This state is characterized by impaired neuromuscular function and loss of ion homeostasis, particularly extracellular ion balance. Upon returning to “normal” temperatures, ion homeostasis recovers and insects regain coordinated movement [48]. Heat knockdown, on the other hand, is cessation of movement due to change of membrane fluidity, ionic balance and structure of macromolecules that influence the rate of metabolic reactions [49].

In this study, we investigate the effects of growth temperature and adult acclimation temperature on thermal tolerance in *T. molitor*. We hypothesise that beetles raised at lower temperatures will show greater tolerance to cold extremes but reduced tolerance to heat extremes, while beetles raised at higher temperatures will exhibit the opposite pattern. These expectations align with the BAH, particularly concerning adult acclimation. Given the implications of climate change and the economic importance of *T. molitor*—both as a pest and as a species bred for food and feed—additional information on its thermal biology could prove critical for future applications.

## 2. Materials and Methods

### 2.1. Experimental Animals

For our experiment, we used the yellow mealworm (*Tenebrio molitor*), an established model species for studies on thermal tolerance [36,39,44]. The beetles were obtained from the Faculty of Natural Sciences and Mathematics colony, where they have been maintained for several years under controlled conditions: a temperature of 22–25 °C, relatively low humidity of 40–50%, and a light period of 12:12 L:D. The larvae and adult beetles were raised on wheat flour and rolled oats, and additionally fed with fresh apples and carrots.

### 2.2. Cold and Heat Tolerance

The last larval stage of *T. molitor* were transferred from colony to Petri dishes and placed in climate-controlled cabinets set to 16, 21, 25, 30 and 35 °C, with ~50% relative humidity. These temperatures were chosen based on the range described in the introduction, which is suitable for mealworm development. Newly pupated individuals were placed in separate Petri dishes and maintained at the same temperature. Upon eclosion, the adult beetles were transferred to plastic containers (8 cm × 15 cm) filled with the same substrate used in the original colony (wheat and oat substrate). Their diet was supplemented with fresh food apples and carrots ad libitum, with any leftover food removed each time fresh food was added to avoid mould growth.

The beetles were observed using a Leica M205C stereomicroscope with a Leica MC 190HD camera attached and LAS X v5.1.0.25593 software (Leica, Wetzlar, Germany) to determine sex according to the methods outlined by Bhattacharya et al. [50]. The weight of the adult beetles was measured as an indicator of body size, with an accuracy of 0.1 mg.

**Figure 1:**
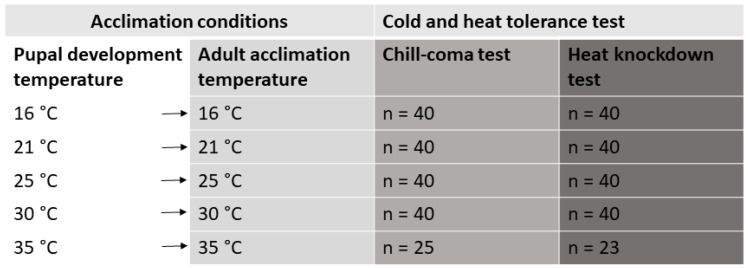
Schematic diagram of the experimental design used to investigate the combined effects of developmental and adult acclimation (five different temperatures) on cold and heat tolerance.

**Figure 2:**
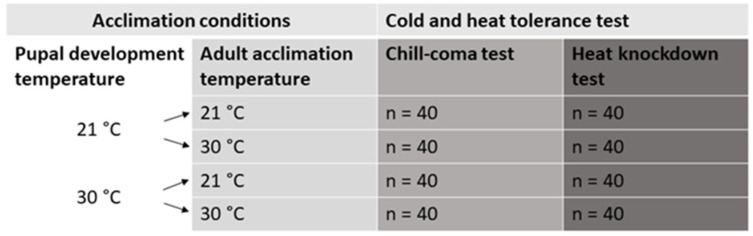
Schematic diagram of the experimental design used to differentiate effects of developmental acclimation and adult acclimation on cold and heat tolerance.

Cold- and heat-shock tests were carried out 1 week after the eclosion, ensuring that all beetles had the same duration (1 week) for adult acclimation. Different individuals were used for the cold- and heat-shock experiments. In the first part of the experiment (Figure 1), we assessed the combined effect of four pupal development temperatures and adult acclimation temperatures (16, 21, 25, 30 and 35 °C) on cold and heat tolerance. In the second part (Figure 2), we aimed to distinguish between the effects of pupal development temperatures and adult acclimation temperatures. For this, 40 individuals from developmental temperatures of 21 and 30 °C were transferred to different adult temperatures to induce acclimation at a temperature different from the developmental temperature during the pupal stage. Another 40 individuals were kept at their original developmental temperature, resulting in a full factorial design with four treatment combinations of developmental and adult acclimation temperatures (2 developmental temperatures and 2 adult temperatures) (Figure 2). Sex ratio was as close to 50:50 as possible, since the number of emerged beetles at certain temperature combinations was limited.

### 2.3. Chill-Coma Test

To induce chill coma, we placed beetles from climate-controlled cabinets into Petri dishes, which were then placed in a water and ice bath to maintain a surface temperature of 5 °C on the Petri dish. The bath temperature was carefully regulated by adding ice cubes and monitoring the surface temperature of the Petri dish with the Fluke 566 Thermal gun infrared and contact thermometer (Fluke Corporation, Everett, WA, USA). The bath temperature was monitored with a classic thermometer. After exactly two hours, the Petri dishes containing the beetles were transferred to a water bath set to 25 °C. We measured the time required for beetles to exhibit coordinated movement of all six legs, similar to the method used by Scharf et al. [51].

### 2.4. Heat-Tolerance Test

For the heat-tolerance test, we conducted a heat knockdown experiment. Beetles were placed in Petri dishes that floated in a temperature-controlled water bath set to 52 °C. The temperature of the Petri dish surface was 44 °C (range of 43.0–44.0 °C), similar to the protocol described in Scharf et al. [8]. The time until the beetles became immobile (complete immobility) was recorded. Immobility was verified by gently touching the beetles with a soft brush, as described in previous studies [8,52,53]. In preliminary experiments, a temperature of 44 °C was found to induce rapid knockdown in *T. molitor* without causing permanent damage, providing sufficient variance in knockdown times between individuals.

### 2.5. Statistical Analysis

For the analysis of chill-coma recovery times, we performed a two-way ANOVA. Combined developmental and adult acclimation temperature and sex, along with the two-way interaction, were used as explanatory variables, and recovery time as the response variable. Tukey post-hoc test was used for pairwise comparison. To distinguish between the effects of developmental acclimation (temperatures during pupal development) and adult acclimation (temperature of adult acclimation), we limited our analysis to beetles that had developed at 21 and 30 °C and were later acclimated to crossed temperatures. A linear model (ANCOVA) was used with developmental temperature and adult acclimation temperature, sex and weight (covariate), along with all two-way interactions as explanatory variables and recovery time as the response variable. Since recovery times were not normally distributed, we applied a log10 transformation.

For analysis of heat knockdown times, we performed Welch’s one-way ANOVA to compare knockdown times between beetles raised at four different temperatures, as the assumption of homogeneity of variances was not met. For pairwise comparison, Games–Howell post-hoc test was used. Additionally, we used Welch’s one-way ANOVA to test the influence of sex on heat knockdown times. To distinguish between the effects of developmental and adult acclimation, we used beetles developed at 21 and 30 °C, which were later acclimated to different temperatures. A linear model (ANCOVA) was used with developmental temperature and adult acclimation temperature, sex and weight (covariate), as well as all two-way interactions as explanatory variables and heat knockdown time as the response variable. All statistical analyses were performed with JASP 0.19.3.

## 3. Results

**Figure 3:**
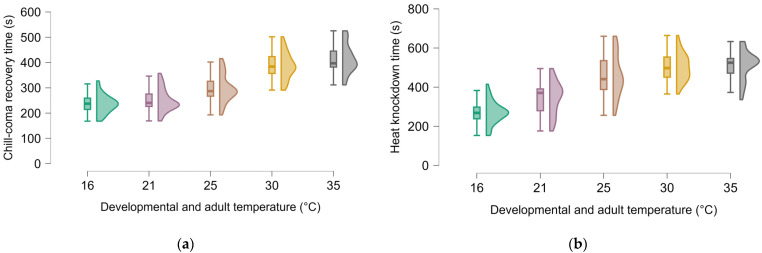
Influence of combined developmental and adult acclimation temperature on chill-coma recovery time (**a**) and heat knockdown time (**b**).

Looking at the effect of temperature on cold resistance without differentiating the effects of developmental and adult acclimation (temperature conditions were not changed in the beetles tested), the beetles responded better to cold exposure when growing at lower temperatures. Chill-coma recovery time was clearly influenced by the combination of developmental and adult temperature (ANOVA: F_4, 175_ = 84.72, *p* < 0.001, µ2 = 0.657 Figure 3a). When the animals were kept at lower temperatures, they recovered faster after the induced chill coma. However, there was no statistical difference between 16 and 21 °C (Tukey test: *p* = 0.506). The recovery times of the groups kept at 16 and 21 °C were shorter than those kept at 25, 30 and 35 °C (Tukey test for all combinations: *p* < 0.001). In addition, the group kept at 25 °C recovered faster than those kept at 30 and 35 °C (Tukey test: *p* < 0.001). However, there was no difference in recovery times between groups kept at 30 and 35 °C (Tukey test: *p* = 0.465). Sex and two-way interaction between sex*growth temperature had no significant impact on chill-coma recovery time. The heat knockdown time was also influenced by the combination of developmental and adult temperature (Welch’s test: F_4, 81.1_ = 87.0 *p* < 0.001, µ2 = 0.574 Figure 3b). Animals kept at higher temperatures were more resistant to heat shock, which means a longer knockdown time. We did not observe a difference between the effect of temperatures of 25 °C and above on heat resistance; however, higher temperatures improved heat resistance in comparison to animals kept at lower temperatures (Games–Howell test: 16 °C < 21 °C < 25 °C = 30 = 35 °C). Sex had no significant effect on heat knockdown times.

**Figure 4:**
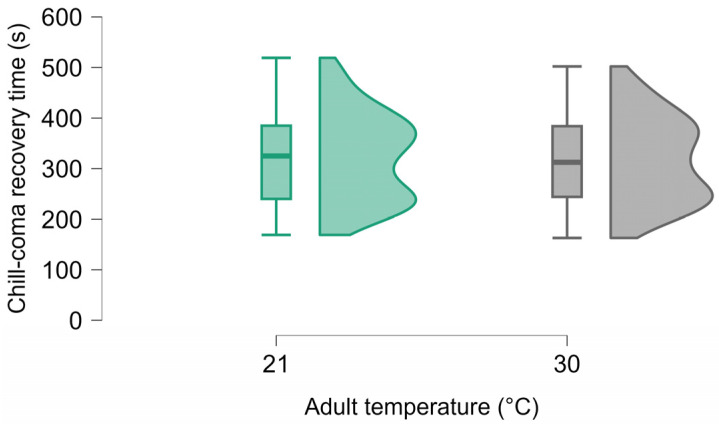
Influence of adult acclimation temperature on chill-coma recovery time.

**Figure 5:**
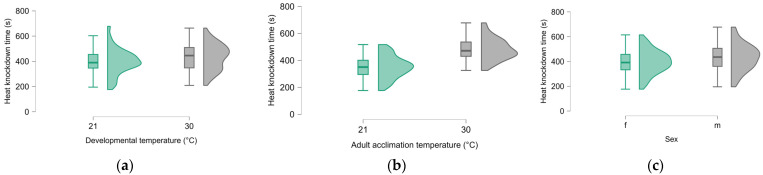
Influence of developmental temperature (**a**), adult acclimation temperatures (**b**) and sex (**c**) on heat knockdown time.

When we attempted to distinguish between the effects of developmental and adult acclimation on chill-coma recovery time, we found that recovery time was only affected by adult acclimation temperature (F_1, 152_ = 344.13, *p* < 0.001, µ2 = 0.687; Figure 4). Beetles acclimated at 21 °C recovered faster than beetles acclimated at 30 °C. Developmental temperature, sex and weight had no effect on recovery time and neither did any two-way interaction. The heat knockdown time was also primarily influenced by adult acclimation temperature (F_1, 142_ = 98.95, *p* < 0.001, µ2 = 0.385; Figure 5b). Beetles acclimated at 30 °C resisted heat shock better than those acclimated at 21 °C. Males resisted heat better than females (F_1, 142_ = 8.19, *p* = 0.005, µ2 = 0.032; Figure 5c). Developmental acclimation temperature (F_1, 142_ = 4.45, *p* = 0.037, µ2 = 0.017; Figure 5a) also had a significant effect on knockdown times with beetles developed at 30 °C resisting heat better than those developed at 21 °C. Weight and two-way interactions had no significant effect on knockdown times.

## 4. Discussion

In our experiment, we aimed to (i) assess the combined effects of growth temperature during pupal and adult stages on cold- and heat-shock resistance, and (ii) investigate the relative importance of developmental acclimation during the pupal stage and adult acclimation on yellow mealworm resilience to temperature extremes.

The first part of the experiment revealed that exposure of *T. molitor* individuals, developing at four different experimental temperatures, to thermal extremes demonstrated that lower growth temperatures enhanced the ability of beetles to resist cold shock, while higher growth temperatures improved heat-shock tolerance. Specifically, beetles raised at 16 and 21 °C, the lowest temperature used in the experiment, exhibited the best resilience to cold shock (measured by chill-coma recovery time) but were most vulnerable to heat shock (measured by heat knockdown time). In contrast, beetles raised at 30 and 35 °C performed best in the heat-shock experiment but were the most susceptible to cold temperatures. A similar acclimation trade-off effect has been reported in *Acheta domesticus* (Linnaeus, 1758) (house cricket), *Tribolium castaneum* (Herbst, 1797) (red flour beetle), *Sarcophaga crassipalpis* Macquart, 1839 (flesh fly), *Drosophila melanogaster* Meigen, 1830 (fruit fly) [8,14,15,54]. These findings support the adaptive nature of acclimation and align with BAH. However, the experimental design of first part of the experiment did not allow us to distinguish between developmental and adult acclimation effects.

Acclimation is a complex process that varies depending on the developmental stage at which it occurs and the length of exposure. At least three distinct forms of acclimation have been recognised [4,5,6,7]. The second part of our experiment focused on determining the relative importance of acclimation at different life stages—pupal and adult. Our results suggest that adult acclimation had the most significant influence on both cold and heat tolerance. Adult beetles acclimated to lower temperatures showed better cold tolerance, while those acclimated to higher temperatures performed better in the heat-tolerance test. Interestingly, the developmental temperatures during the pupal stage also contributed to resistance, particularly in the heat-tolerance test. However, pupal stage temperatures had no effect on cold-shock resistance, as indicated by chill-coma recovery time. These results provide partial support for the beneficial acclimation hypothesis (BAH) and are partly consistent with Scharf et al. [8], who observed a similar effect in *Tribolium castaneum*. In their study, beetles acclimated as adults to lower temperatures resisted cold shock better, while those acclimated on higher temperatures showed the opposite. However, their results differ from ours in that they found a positive effect of higher developmental temperatures on cold tolerance, which we did not observe in our experiment.

The BAH proposes that individuals that develop at a specific temperature perform better at that specific temperature compared to individuals that developed at other temperatures [16,20]. While studies show mixed support for this hypothesis, one possible explanation for its lack of support is that long-term exposure to non-optimal conditions during developmental life stages could impair overall fitness, thus decreasing performance in all subsequent environmental conditions. Nevertheless, our results show that development at higher temperatures did enhance heat tolerance. We can argue that this shows partial support for BAH. In contrast, other authors propose the optimal development temperature hypothesis [17,18,19,22], which suggests that organisms raised at intermediate (optimal) temperatures perform better across all temperatures compared to those raised at higher or lower temperatures. Two more hypotheses are proposed, called the “warmer is better” and “colder is better” hypotheses. They predict higher relative fitness at all temperatures for organisms raised either at warm temperatures (“warmer is better”) than by organisms raised at low and intermediate temperatures [8,55] and vice versa for the “colder is better” hypothesis [7,18,30]. Our results from the second experiment do not unambiguously support either of these alternative hypotheses. We can clearly reject the “colder is better” hypothesis, as development at 21 °C did not improve thermal tolerance to either low or high temperatures. Since we used 30 °C as the optimal developmental temperature and 21 °C as a suboptimal temperature, we can argue, similarly with support for the BAH, that the results partially support the “optimal is better” hypothesis and even the “warmer is better” hypothesis. However, when combining the findings from both parts of the experiment and looking at the low-temperature acclimation effect on cold resistance, we can see that insects kept 16 °C did not outperform those kept at 21 °C in the cold-tolerance test. This is true for the heat-tolerance test as well; insects kept at 35 °C did not perform better than those kept at 30 °C. The first experiment supplements our findings in favour of the “optimal is better” hypothesis. Still further testing is needed to unambiguously confirm or challenge this hypothesis and to explore the effect of suboptimal developmental temperatures on stress tolerance.

In the second part of the experiment, we also observed that males tolerated heat better than females, although there was no sex-related effect on cold tolerance. Scharf et al. [8] report similar findings for *T. castaneum*. The influence of sex on thermal tolerance is not well understood [56]. In studies of acclimation, sex is often considered as an explanatory variable, though it is typically not the primary focus. The results on this topic reported in different studies are therefore ambiguous, with varying outcomes [8,45,57,58].

In summary, acclimation is a complex process occurring at different stages of an organism’s life cycle, and according to our results, it has an adaptive nature. Different forms of acclimation contribute to overall fitness under altered temperature conditions. Our study focused on two forms of acclimation—developmental and adult—but did not address hardening. Our results indicate that adult acclimation plays the predominant role in mealworm adjustments to unfavourable conditions, particularly in cold extremes. In hot conditions, yellow mealworms also rely on developmental temperatures, with higher growth temperatures contributing to better performance at high temperature extremes. In this study, we did not examine the influence of temperature fluctuations during the development and acclimation process, which can impact development and stress tolerance in insects [59,60]. This is a topic worthy of further investigation. For future research, we also propose a study that would give more definitive answers regarding the contributions of different forms of acclimation to thermal tolerance. Given the species’ widespread distribution and increasing economic importance, we suggest continued use of *T. molitor* as a model species for studies in insect thermal biology.

## Data Availability

Data is contained within the article and supplementary material.

## Supplementary Materials

The following supporting information can be downloaded at: https://www.mdpi.com/article/10.3390/insects16040402/s1.

## Abbreviation

The following abbreviation is used in this manuscript:

BAH	Beneficial acclimation hypothesis
